# Types and cost of wellness services provided by a neighborhood-based academic nursing center

**DOI:** 10.3389/fpubh.2024.1477436

**Published:** 2025-01-08

**Authors:** Hae-Ra Han, Yoon-Jae Lee, Jennifer J. Lee, Chun-An Sun, Ophelia Dapaah-Gyimah, Patty Wilson, Catherine Ling, Eric Slade

**Affiliations:** ^1^Johns Hopkins University School of Nursing, Baltimore, MD, United States; ^2^Johns Hopkins University Bloomberg School of Public Health, Baltimore, MD, United States; ^3^University of Pennsylvania School of Nursing, Philadelphia, PA, United States; ^4^Leonard Davis Institute of Health Economics, University of Pennsylvania, Philadelphia, PA, United States

**Keywords:** community center, wellness services, vulnerable population, nurses, cost

## Abstract

**Background:**

Despite increased insurance coverage since 2010, racial and ethnic minorities in the United States still receive less medical care than White counterparts. The Johns Hopkins School of Nursing’s Center for Community Programs, Innovation, and Scholarship (COMPASS Center) provides free wellness services, aiming to address healthcare disparities in the neighborhoods.

**Objective:**

To delineate the types and cost of wellness services provided by the COMPASS Center.

**Methods:**

The study employed a secondary analysis design, utilizing Qualtrics surveys to assess wellness service data from 2017 to 2022 at two main program sites—Wald center and House of Ruth, Maryland.

**Results:**

The analysis covered 2,194 encounters (826 at Wald center and 1,368 at House of Ruth, Maryland). Most encounters at both sites served African American/African/Black and low-income individuals. Examples of wellness services included pre-employment exams and immunizations, health literacy and self-care management education, health insurance advice, parenting support, and referrals to community resources. Cost analysis revealed varying expenses per encounter, with medium costs ranging from $5.45 to $14.91 across sites, considering service type and duration, including staff salaries, encounter numbers, and service hours.

**Conclusion:**

The COMPASS Center delivers essential wellness services supplementing traditional healthcare to disadvantaged community members through student engagement and academic support. The next generation of healthcare teams is learning with and from the community creating a holistic educational experience in building skills outside of institutional bedsides. Future plans involve structured training for students and center staff to provide wellness services, while expanding social service referrals. More work is needed to evaluate the impact of our wellness services on client satisfaction and wellness improvements.

## Background

The healthcare model in the United States (U.S.) is diverse and dynamic with a mix of providers as well as private and public insurers. Since the enactment of the Affordable Care Act in 2010, the national uninsured rate has decreased from 16 to 8.5% (1). While governmental efforts have improved overall coverage rates in the U.S. population, this does not necessarily reflect equal access to healthcare. Racial and ethnic disparities are particularly pronounced, with studies showing that racial and ethnic minority groups often face limited access to care, lower quality of care, and, consequently, worse health outcomes compared to White individuals (2–4). These disparities are partly due to a number of institutional barriers, such as racial biases and the unequal distribution of the healthcare budget (5, 6). As the U.S. population becomes increasingly diverse, with projections indicating that people of color will represent over half of the population by 2050 (2), addressing these disparities has become a priority.

In an effort to mitigate these inequalities, community-level initiatives, such as federally qualified health centers and free clinics, have been shown to positively impact health outcomes among vulnerable populations (7). Acknowledging the impact of these community-centered efforts, the Johns Hopkins School of Nursing has made significant investments in the neighborhood, fulfilling part of its mission to promote ‘the health of individuals and diverse communities locally.’ In 1994, the school established the Lillian C. Wald Community Wellness Center to provide health screenings, health education and counseling, vaccinations, and referrals at no charge to low-income, uninsured, or underinsured residents (referred to as ‘wellness services’ hereafter). Since then, the school has expanded its community-based health promotion programs to include additional sites such as House of Ruth Maryland—a transitional housing facility for women escaping from domestic violence—and Northeast Market—a neighborhood market selling fresh products and other goods—in Baltimore, Maryland.

Currently, the school’s Center for Community Programs, Innovation, and Scholarship (COMPASS Center) serves as an operational umbrella for the school’s community wellness service initiatives (8). One unique aspect of COMPASS Center initiatives is that all activities are delivered by nursing students and nurses from the school’s academic programs (e.g., master’s entry into nursing, DNP and PhD programs), under the supervision of nursing faculty. One unique aspect of COMPASS Center initiatives is that all activities are delivered by nursing students and nurses from the school’s academic programs (e.g., master’s entry into nursing, DNP, and PhD programs), supervised by nursing faculty. This student-centered approach has proven to be a highly effective model for delivering services to communities (9). Although similar wellness programs are offered by other academic institutions (9, 10), our program provides a broader range of wellness services, extending beyond basic health screenings to include support for women experiencing domestic violence.

Given our sustained efforts to promote health and wellness among underserved individuals in the community, it is important to understand the impact of our work. While this program was intended to provide free healthcare services without profit, understanding its costs and benefits allows us to evaluate its impact on the community and assess the efficacy of the program model in comparison to alternatives (11–13). Therefore, the purposes of this analysis were two-fold: (1) To describe the types and nature of health and wellness services provided to the community, and (2) To estimate the cost associated with providing these services.

## Methods

### Design and data sources

The study design was a secondary analysis of the wellness service data captured through Qualtrics surveys. The first time a Qualtrics survey was created to enter wellness service-related encounters was in 2017. Since then, additional Qualtrics surveys were created for different years, sites, and initiatives. We pooled all available Qualtrics surveys at the time of this analysis, covering encounter data collected from January 2017 to March 2021 on the clients served at the Lillian C. Wald Community Wellness Center (Wald Center hereafter) (*n* = 1,382), and from January 2017 to March 2022 on the clients served at the House of Ruth Maryland (*n* = 1,378), yielding a total of 2,760 clients across sites (Note: Anonymous data is available upon request through https://livejohnshopkins.sharepoint.com/:x:/r/sites/SONCCIAS/_layouts/15/Doc.aspx?sourcedoc=%7BBDA248C7-3BD5-41E9-BB73-D6AADF2E89F0%7D&file=Wald_Clean.xlsx&action=default&mobileredirect=true).

### Procedures

Upon the approval by the Johns Hopkins Medicine Institutional Review Board, the study team pooled the raw community health and wellness service data from the Qualtrics server. The Qualtrics surveys included questions about the date of service, client age, gender, race/ethnicity, medical history, any health problems addressed during the encounter, types of visits and services received, assisting staff during the encounter, and any referrals made. These surveys were completed by assisting staff (e.g., nursing student, licensed registered nurse, or community health worker) at the time of the visit. No personal identifiers were collected. Additionally, the team reached out to several faculty and staff who had working knowledge and experiences with wellness services offered by COMPASS Center to clarify types and nature of services while obtaining further details about staff who provided such services (e.g., nursing student, licensed registered nurse, or community health worker) or estimated time spent on each type of services as such level of detailed information was not collected as part of the encounter survey at the time of this analysis.

### Analysis

Due to different types of wellness services provided, each site coded service encounters differently in the Qualtrics surveys. The team created shared data coding between the two sites to create a pooled dataset. After creating the pooled and cleaned dataset, we used descriptive statistics to summarize client characteristics and counts of wellness services. Of note, 486 of the 1,382 clients recorded at the Wald Center were research participants who came in for in-person data collection. Additionally, 80 clients (70 for Wald and 10 for House of Ruth Maryland) did not record encounter types and/or nature. After removing them, the final dataset included data from a total of 2,194 clients (*n* = 826 for Wald and *n* = 1,368 for House of Ruth Maryland).

For this cost analysis, the study team created a new variable—time spent on each service. The team used ranges of time spent on each service (as opposed to specific time values, unless standard time values were available) based on COMPASS Center faculty and staff recollection. The time range for each type of service is described in Tables 1, 2. For analysis purpose, if an encounter time range was 10–15 min, we assigned 10, 12.5, and 15 min for low, medium, and high time points, respectively. All minutes were then converted to hours to generate costs per service based on the hourly salary of the staff person who provided the service. Staff who provided wellness services were registered nurses and a community health worker at the school. The estimated hourly wage for community registered nurse was $28.58 per hour, which corresponds to the national mean wage for registered nurses reported in the 2021 U.S. Bureau of labor statistics national occupational wage estimates (14). The hourly wage of a community health worker was estimated as $20.83 per hour. Cumulative total cost per service was calculated by multiplying service encounter hours for each service type by the corresponding hourly wage. The average cost per encounter was estimated by total cost divided by total encounters.

**Table 1:** Estimated costs for services provided at Wald Center.

Program	Time range (hour)	Encounter	Time spent per encounter			Total service hours			Total cost			Average cost per encounter		
			Low	Medium	High	Low	Medium	High	Low	Medium	High	Low	Medium	High
PPD (first visit)	0.25	182	0.25	0.25	0.25	45.5	45.5	45.50	$1,300.39	$1,300.39	$1,300.39	$7.15	$7.15	$7.15
PPD (follow-up)	0.05–0.08	155	0.05	0.07	0.08	7.75	10.33	12.92	$221.50	$295.33	$369.16	$1.43	$1.91	$2.38
Ask A nurse	0.25	5	0.25	0.25	0.25	1.25	1.25	1.25	$35.73	$35.73	$35.73	$7.15	$7.15	$7.15
Follow up	0.05–0.08	106	0.05	0.07	0.08	5.30	7.07	8.83	$151.47	$201.97	$252.46	$1.43	$1.91	$2.38
Work/School physical exam	0.5–0.75	32	0.5	0.63	0.75	16.00	20	24.00	$457.28	$571.60	$685.92	$14.29	$17.86	$21.44
Immunization	0.25	9	0.25	0.25	0.25	2.25	2.25	2.25	$64.31	$64.31	$64.31	$7.15	$7.15	$7.15
Labs	0.17	29	0.17	0.17	0.17	4.83	4.83	4.83	$138.14	$138.14	$138.14	$4.76	$4.76	$4.76
Appointment scheduling/Accompaniment	0.25–0.5	44	0.25	0.38	0.50	11.00	16.5	22.00	$229.13	$343.70	$458.26	$5.21	$7.81	$10.42
General wellness counseling	0.17–0.25	246	0.17	0.21	0.25	41.00	51.25	61.50	$1,171.78	$1,464.73	$1,757.67	$4.76	$5.95	$7.15
Phone consult	0.17	18	0.17	0.17	0.17	3.00	3	3.00	$85.74	$85.74	$85.74	$4.76	$4.76	$4.76
Total		826				137.88	161.98	186.08	$3,855.46	$4,501.61	$5,147.76	$4.67	$5.45	$6.23

PPD = purified protein derivative (PPD) skin test for tuberculosis; Ask A Nurse = phone-based service that provided SDOH screenings, general health information, and local community resources; Follow-up = Follow up for any of the services offered, which includes ‘Ask A Nurse,’ ‘General wellness counseling’, or basic laboratory tests results, Work/School physical exam = exams required for work/school, immunizations for seasonal influenza, tetanus, MMR (adults only); Labs = urine analysis, comprehensive blood count, and basic metabolic panel; Appointment scheduling/Accompaniment = a community health worker provided assistance with making medical appointments and also accompanied people to appointments; General wellness counseling = counseling on nutrition, physical activity, and sleep; general wellness counseling; Phone consult = phone-based provision of health education on SDOH or medical conditions.

**Table 2:** Estimated costs for services provided at House of Ruth Maryland (*n* = 1,368).

Type of visit	Time range (hour)	Encounter	Time spent per encounter			Total service hours			Total cost			Average cost per encounter		
			Low	Medium	High	Low	Medium	High	Low	Medium	High	Low	Medium	High
Office visit (public space)	0.33–1	25	0.33	0.67	1.00	8.33	16.67	25	$238.17	$476.33	$714.50	$9.53	$19.05	$28.58
Office visit (private space)	0.25–1.5	527	0.25	0.875	1.5	131.75	461.125	790.5	$3,765.42	$13,178.95	$22,592.49	$7.15	$25.01	$42.87
Telephone visit	0.25–0.5	588	0.25	0.375	1	147	220.5	294	$4,201.26	$6,301.89	$8,402.52	$7.15	$10.72	$14.29
Home visit	0.25–0.75	112	0.25	0.5	0.5	28	56	84	$800.24	$1,600.48	$2,400.72	$7.15	$14.29	$21.44
Group activity	60	32	60	60	0	60	60	60	$1,714.80	$1,714.80	$1,714.80	$53.59	$53.59	$53.59
Text	0.08–0.17	358	0.08	0.125	0.75	29.83	44.75	59.67	$852.64	$1,278.96	$1,705.27	$2.38	$3.57	$4.76
Total		1647*				404.92	859.04	1313.17	$11,572.52	$24,551.41	$37,530.30	$7.03	$14.91	$22.79

*Multiple entries per client were allowed at this site, hence the resulting total encounter number is greater than the number of clients served. Clients were provided services in multiple locations, so visit types were organized accordingly. Public office visit = meeting clients in public settings within HRM such as the cafeteria, hallway, computer room, etc.; Private office visit = meeting clients at the health suite; Telephone visit = calling clients to meet their needs (i.e., assistance with making health appointments, signing up for insurance, medicine fulfillments, etc.); Home visit = Meeting clients in their personal rooms in the shelter; Group activity = health promotion/education activities with multiple clients; Text: texting clients to communicate and meet health needs.

## Results

### Characteristics of clients

The Wald Center and the House of Ruth Maryland served different clients and naturally, provided different types of wellness services. At the Wald Center, the majority of clients were adults aged between 25 and 34 years (23%). More women (64.2%) sought wellness services compared to men (32.8%). The majority of people were identified as African American/African/Black (88.9%), and as non-Hispanic or Latino (90.3%). Nearly half (47.1%) were uninsured and 17.3% had Medicaid. At House of Ruth Maryland, clients were often between the ages of 25 and 34 (40.4%) and almost all of the clients served were female (91.5%). More than two thirds of the clients identified as African American/African/Black (74.3%). About 69.5% of House of Ruth clients were unemployed and 68.1% had Medicaid (Table 3).

**Table 3:** Wellness service client characteristics.

	Cross-site (*N* = 2,194)	Wald (*n* = 826)	HRM (*n* = 1,368)
Age, *n* (%)			
<18	211 (9.6)	8 (1)	203 (14.8)
18–24	240 (10.9)	101 (12.2)	139 (10.2)
25–34	742 (33.8)	190 (23)	552 (40.4)
35–44	414 (18.9)	113 (13.7)	301 (22)
45–54	243 (11.1)	102 (12.3)	141 (10.3)
55–64	159 (7.2)	132 (16.0)	27 (2)
65+	133 (6.1)	133 (16.1)	0
Missing	52 (2.4)	47 (5.7)	5 (0.4)
Gender, *n* (%)			
Male	384 (17.5)	271 (32.8)	113 (8.3)
Female	1782 (81.2)	530 (64.2)	1,252 (91.5)
Transgender	2 (0.1)	0	2 (0.1)
Missing	26 (1.2)	25 (3)	1 (0.1)
Race, *n* (%)			
African American/African/Black	1751 (79.8)	734 (88.9)	1,017 (74.3)
Native Hawaiian/Pacific Islander	8 (0.4)	0	8 (0.6)
Asian	23 (1)	18 (2.2)	5 (0.4)
White/Caucasian	263 (12)	16 (1.9)	247 (18.1)
American Indian/Alaska Native	1 (0.05)	1 (0.1)	0
More than one race	4 (0.2)	1 (0.1)	3 (0.2)
Other	87 (4)	22 (2.7)	65 (4.8)
Missing	57 (2.6)	34 (4.1)	23 (1.7)
Hispanic or Latino, *n* (%)			
Yes	129 (5.9)	22 (2.7)	107 (7.8)
No	1904 (86.8)	746 (90.3)	1,158 (84.6)
Missing	161 (7.3)	58 (7)	103 (7.5)
Employment, *n* (%)			
Unemployed	–	–	951 (69.5)
Employed full-time	–	–	88 (6.4)
Employed part-time	–	–	40 (2.9)
Seasonal	–	–	9 (0.7)
Missing	–	–	280 (20.5)
Health insurance, *n* (%)			
Medicaid	1,075 (49)	143 (17.3)	932 (68.1)
Medicare	142 (6.5)	101 (12.2)	41 (3)
Veteran affairs	14 (0.6)	14 (1.7)	0
Private insurance	59 (2.7)	2 (0.2)	57 (4.2)
Affordable care act	3 (0.1)	0	3 (0.2)
Uninsured	541 (24.7)	389 (47.1)	152 (11.1)
Insurance pending	31 (1.4)	20 (2.4)	11 (0.8)
Other	24 (1.1)	24 (2.9)	0
Missing	305 (13.9)	133 (16.1)	172 (12.6)

### Description of types and nature of wellness services

Tables 1, 2 provide lists of services offered at Wald Center and House of Ruth Maryland, respectively. The types of services offered at Wald Center mainly consisted of pre-employment physical exams and vaccinations, whereas health maintenance and education were prioritized at the House of Ruth Maryland.

There were nine types of services provided at Wald, which included purified protein derivative (PPD) skin test for tuberculosis and/or follow-up (for reading of the skin test result), Ask A Nurse (a phone-based service that provided social determinants of health screening and general health information as well as connecting patients with local community resources), general follow-up for any of the services offered at the center, work/school physical examinations, immunizations for seasonal influenza, tetanus, and Measles, Mumps, and Rubella (MMR) (adults only), basic laboratory tests, medical appointment scheduling, general wellness counseling, and phone consultations. Basic laboratory tests (e.g., urine analysis, comprehensive blood count, and basic metabolic panel) were available for those who came for an initial physical examination. The appointment scheduling service was led by a community health worker who accompanied the patients to their necessary appointments for medical and/or social services. General wellness counseling embraced nutrition, physical activity, and sleep. Lastly, phone consultations were used to provide health education and referrals to address social determinants of health or ongoing medical care for patients.

At the House of Ruth Maryland, services were rendered in group and individual settings by community health nurses who operated the health suite within the transitional shelter. The nurses met with clients within the health suite, in public areas within the shelter, via phone or text communication, and in residents’ rooms. The nurses addressed episodic health concerns by conducting blood pressure and blood sugar screenings, providing over-the-counter medications per protocol or consultations for over-the-counter medications that were available on-site (e.g., pain or allergy relief medications, cortisone, calamine lotion, antacids, multi- and prenatal vitamins, and antifungal medications). The health suite had medications for both adult and pediatric populations. To address episodic concerns among clients at the House of Ruth, the nurses also administered seasonal flu vaccines and conducted physical exams to assess symptom acuity and need for referral to primary care or emergency care services. Health assessments were also performed on children in need of daycare and childcare approval. The nurses also assisted families with accessing pharmacies, obtaining health insurance, and connected them to primary care and other necessary health services. The nurses also facilitated “Health Talks” on different health topics such as self-breast examinations, nutrition, mental health, and personal hygiene to educate and promote the health of shelter clients.

### Estimated costs associated with wellness services

Tables 1, 2 also outline the estimated costs associated with each type of visit, categorized by low, medium, and high ranges of service times at both sites. Services at the Wald Center were provided by community registered nurses and a community health worker to a total of 826 individuals, incurring a total cost ranging from $3,855.46 to $5,147.76, with the average cost per encounter across the various programs ranging from $4.67 to $6.23. For the PPD, patients completed intake forms and skin tests during the initial encounter and returned for result follow-ups. Initial visits (*n* = 182) lasted 15 min, while return visits (*n* = 155) lasted 3–5 min. Initial encounters cost $7.15, while subsequent visits ranged from $1.43 to $2.38 per encounter. The work/school physical exam required the longest service time at 30–45 min among the various programs. Despite the relatively small number of encounters (*n* = 32), this service had the highest average cost per encounter, ranging from $14.29 to $21.44. General wellness counseling had the highest total encounters (*n* = 246), with a cost per encounter ranging from $4.76 to $7.15 due to its shorter service time. Appointment scheduling had the second-highest cost at $10.42 per encounter. Lastly, despite the differences in encounter numbers and service durations, the immunization service, appointment scheduling, and Ask A Nurse program had the same average cost per encounter at $7.15. Similarly, labs and phone consult showed an identical cost per encounter at $4.76.

In contrast to Wald where single entry per encounter was recorded, multiple entries were allowed per encounter at the House of Ruth Maryland. Consequently, the number of encounters recorded (*n* = 1,647) was larger than the number of clients served at this site (*n* = 1,368). The total costs were estimated at $11,572.52 for the low estimated time, $24,551.41 for the medium time, and $37,530.30 for the high range of hours. The average costs per encounter across the various programs at this site ranged from a low of $7.03 to a high of $22.79. Looking at each program, community nurses provided office visits either in a public space or in a private space. For office visits in a public space, the service time per encounter was estimated to be between 20 and 60 min. With 25 encounters, the estimated costs ranged from $238.17 (low) to $714.50 (high). The mean cost per encounter was $9.53 (low), $19.05 (medium), and $28.58 (high) within each time range. On the other hand, office visits in a private space had a higher encounter count (*n* = 527) with a time range of 15 to 90 min. The total estimated cost for these encounters varied from $3765.42 to as high as $22,592.49. The average cost per encounter spanned from $7.15 to $42.87. Similar to office visits, home visits also addressed individual needs, with 112 recorded encounters. The total estimated cost for home visits varied between $800.24 and $2,400.72. The average cost per encounter ranged from $7.15 to $21.44. Telephone visits, highly demanded service with the highest encounter numbers (*n* = 588), had a time range of 15–30 min per encounter. The total estimated cost for telephone visits ranged between $4,201.26 and $8402.52. Despite the high encounter numbers, the relatively short encounter type resulted in an average cost per encounter ranging from $7.5 to $14.29. This site also provided group activity which involved 32 individuals, accounting for a total of 60 h. The average cost per encounter for group activities was $53.59. Counseling services through text messages, with a brief duration of 5–10 min per communication, totaled 358 encounters; the average cost per encounter ranged from $2.38 to $4.76.

## Discussion

The current analysis revealed that a neighborhood-based nursing center affiliated with an academic institution has provided a wide ranges of wellness services to individuals in the neighborhood, most of whom were African American or Black, low-income, uninsured, and unemployed. It is particularly important to address the needs of these populations, as prior studies have shown that Black individuals experience a higher prevalence of lack of health insurance and unmet health needs compared to White individuals, due to both structural and racial/ethnic disparities (15). The historical segregation of minority groups across the U.S. means that Black individuals are more likely to live in medically underserved areas, where quality care is often unavailable, creating significant barriers to essential primary care services (16). By engaging nursing students and licensed registered nurses in the academic programs as the main providers of the wellness services, the costs were estimated to be low, with medium costs per encounter of $5.45 and $14.91 at Wald Center and House of Ruth Maryland, respectively.

By way of providing health screening and other types of wellness programs to local residents, our community nurses had a first-hand opportunity to understand and address the health needs of the community. The provision of service-learning activities has been noted as an innovative strategy for providing nursing and allied health students with a population perspective of health (17–19). Other community-oriented service models have been reported. For example, the mobile clinic model has served high-needs populations by offering health outreach, harm reduction services, and referrals at convenient community locations (20, 21). Our service model is academic center-based registered nurses who are in the graduate programs and are supervised by nursing faculty with advanced practice credentials and doctoral degrees. Student engagement in this type of service enhances empathy, knowledge, and the intent to practice in underserved populations—key aspects of future healthcare professionals’ development (10). One study also estimated the educational value provided to students in a student-run free clinic at $73,751 annually (22). Such service not only benefits the community but also provides significant value to the school. Research is warranted to investigate student perspectives and experiences to work as community nurses and how it impacts their orientation and skills to act as health agents in promoting health equity.

One of the key services offered was PPD testing. PPD testing (for Tuberculosis infection) is recommended or required for individuals who live or work in high-risk settings such as correctional facilities, nursing homes, or homeless shelters and healthcare workers (23). While it was not clearly documented in the Qualtrics forms as to in what context PPD testing was requested, it is likely that the testing was done as part of pre-employment health screenings as these tests were all onsite-based. Decades of research support the relationship between employment and better health (24). Individuals with low incomes have worse health status than the general U.S. population (25), due to limited or lack of resources that could bolster their health such as access to health insurance and necessary healthcare (24, 26). Pre-employment health screening is an essential step for certain individuals to whom it is required. Neighborhood-based pre-employment health screening such as ours may help promote employability among low-income and unemployed people who otherwise may not have access to such services. Future research should investigate if community-based nurse-led pre-employment health screening can affect individual’s economic viability through employment and subsequent health outcomes.

More than two thirds of the encounters at the House of Ruth Maryland were private office visits or phone visits where one-on-one services were provided. Similar to the wellness services offered at the Wald Center, the one-on-one services offered on site encompassed health screenings (e.g., PPD) and immunizations (e.g., hepatitis B) that are often required for employment (27). Of note, at this particular site, a homeless shelter and transitional housing facility for both women and their children escaping violence, our nurses also provided health assessments for children in need of daycare and childcare approval. Transitional or temporary housing offers people experiencing homelessness with interim housing stability and supportive services such as life skills training, mental health services, legal services, and employment assistance (28). Prior research has primarily addressed the outcomes of housing interventions on mental health, safety, and stress among women experiencing intimate partner violence (29). In contrast, the evidence gaps in investigating outcomes related to their children and/or how different types of supportive services are associated with long-term outcomes for these women such as employment. Financial stability was associated with higher odds of survivors transitioning into the ‘doing better’ class (as opposed to being in the ‘high abuse’ or ‘still affected’ class) over 6 months among survivors seeking services from domestic violence agencies (30). Taken together, it would be important to understand the impacts our wellness services may have on the lives of women and children in transitional housing.

Healthcare expenses are a major barrier to accessing care services. Policy changes that directly address the financing challenges faced by marginalized populations face are imperative. While the Affordable Care Act has provided much-needed insurance support to many, additional policies are needed to expand insurance plan coverage and advance home-based care services, making healthcare more affordable (31). Furthermore, care models that bring health services directly to the community, such as the efforts at the Wald Center and the health suite at the House of Ruth, may help make healthcare more available and accessible to those who need it most.

### Limitations

The encounter data did not have specific information addressing time spent on each service. To enable comprehensive cost estimation, we created three different cost estimation models based on faculty and staff recollections. While those with prior knowledge and experience with the wellness services offered by COMPASS Center generally provided uniform responses, their recollections may have been influenced by recall bias. The dataset was also cross-sectional in nature. Whether these encounters resulted in health outcome changes remained unknown. It would be important to evaluate program outcomes in terms of increasing linkage to primary care services, emergency department overuse avoidance and delivering essential supplies, as has been reported for mobile clinics (21, 32). Additionally, we did not have access to data on non-personnel costs associated with wellness service encounters. A report commissioned by the Health Resources and Services Administration found that non-personnel costs at urban public health clinics accounted for approximately one-third (33.2%) of total healthcare delivery costs in 2021 (33). Based on this percentage, we estimate that our calculations may underestimate the total cost of student nurse encounters by approximately 50%. Lastly, the data were derived from one institution in a particular geographic area, limiting generalizability of the findings.

## Conclusion

The COMPASS Center has provided wellness services to low-income, un/under-insured, and unemployed individuals. We found that the types and nature of services mainly included pre-employment physical exams, vaccinations, and health maintenance and education, with medium costs ranging from $5.45 to $14.91. Lack of trust being an independent factor in delays seeking health care (34, 35), our ongoing presence in the community might help build trust with the institution. To this end, our neighborhood-embedded center leveraging students in health professional programs may serve as a model to supplement the traditional health and social care systems for individuals and communities who—due to issues of trust and/or income, language, location, residency status—are “medically disenfranchised” (36). Since the COVID-19 pandemic, our team worked hard to recover and regain our service capacity by hiring new staff, while reconnecting with our partners. As we continue strengthening our endeavors, we envision our student and staff training will be more structured and expanded to allow further engagement with social service sectors, while continuing to promote coordinated primary care referrals and wellness services.

## Data Availability

The data analyzed in this study is subject to the following licenses/restrictions: the anonymous data used for this analysis will be made available upon reasonable request. Requests to access these datasets should be directed to hhan3@jhu.edu.
